# Supplementary material for: Diarrhea prevalence in a randomized, controlled prospective trial of point-of-use water filters in homes and schools in the Dominican Republic
Source: Trop Med Health. 2021 Jan 4;49:1. doi: 10.1186/s41182-020-00291-y (PMC7780396; doi:10.1186/s41182-020-00291-y)
Supplement: Supplementary file 3 — Additional file 3. Prevalence of self-reported diarrhea by home filter status: Intent to Treat Analysis. [file 41182_2020_291_MOESM3_ESM.pdf]

Additional File 3. Prevalence of self-reported diarrhea by home filter status: Intent to Treat Analysis

**Aggregation**	**Before filter in home**[1] % (x/n)	**After filter in home**[1] % (x/n)	**OR (95% CI)**[2]	**aOR (95% CI)**[3]
Any member of the household	25.6% (101/394)	10.3% (70/681)	0.26 (0.17,0.383)***	0.31 (0.18,0.52)***
Adults only	17.3% (68/392)	4.8% (32/666)	0.07 (0.03,0.16)***	0.26 (0.13,0.53)***
School aged children only	12.2% (46/378)	5.7% (37/648)	0.22 (0.09,0.5)***	0.41 (0.19,0.86)*
Children less than 5	11.8% (12/102)	7.1% (12/169)	0.50 (0.18,1.37)	0.47 (0.12,1.83)

*$p<0.05$; **$p<0.01$, ***$p<0.001$*

[1]Counts are each measurement of each household and include households reporting no filter usage

[2]Odds Ratio accounting for repeated measures

[3]Adjusting Odds Ratio accounting for repeated measures and adjusted for Household size, Season (Fall, Winter, Spring), Water Source, School Filter Status and Region
